# Therapeutic and Predicted Significance of PKLR in Patients With Cholangiocarcinoma

**DOI:** 10.1002/cnr2.70649

**Published:** 2026-08-14

**Authors:** Biyan Gong, Xuejun Xu, Tubing Xu, Dong Zhang, Li Yang, Cailing Li, Tingjun Li, Yuandeng Luo

**Affiliations:** ^1^ Department of General Surgery Armed Police Corps Hospital Chongqing China; ^2^ Department of Hepatobiliary Surgery General Hospital of Xinjiang Military Command Xinjiang China; ^3^ Department of Thyroid and Breast Surgery General Hospital of Xinjiang Military Command Xinjiang China; ^4^ Department of Nursing General Hospital of Xinjiang Military Command Xinjiang China

**Keywords:** cholangiocarcinoma, nomogram, overall survival, PKLR, prognosis

## Abstract

**Background:**

Cholangiocarcinoma (CCA) is a highly heterogeneous malignancy with a poor prognosis. However, the molecular mechanisms underlying CCA and its treatment options remain unclear and limited.

**Aims:**

This study aims to identify novel prognostic biomarkers and potential therapeutic targets for CCA through transcriptomics and whole‐exome sequencing analysis.

**Methods and Results:**

We analyzed the relationship between overall survival and various risk factors in 255 CCA samples from a published dataset and 36 samples from The Cancer Genome Atlas (TCGA) database. Transcriptomics analysis was used to identify genes whose expression plays a crucial role in CCA development and progression. Targeted drug databases were used to examine which gene expression signatures represent potential therapeutic targets for CCA. Mutated genes were analyzed to explore potential mechanisms. Standard statistical methods were applied to analyze associations between clinical data and candidate targets. A nomogram was constructed to predict the prognostic impact of the optimal candidate. Cell experiments were used to demonstrate the role of PKLR in CCA growth and metastasis. We identified FOLH1, PKLR, PTH1R, GPR114, KCNH2, LCN2, MMP10, PORCN, RIPK3, and SRPK3 as potential therapeutic targets for CCA, with PKLR being the most promising candidate based on the computational analyses. PKLR was lowly expressed in CCA tissues. PKLR expression was closely associated with ARID1A and KRAS mutations. PKLR mRNA levels were significantly correlated with tumor size (*p* = 0.024) and metastasis (*p* = 0.014). Low PKLR expression in CCA was positively associated with poor survival. A nomogram accurately predicted the prognosis of CCA patients based on PKLR expression levels. Silencing PKLR expression promotes growth and metastasis of CCA in vitro.

**Conclusion:**

Our findings indicate that PKLR, an independent protective factor in CCA, is a potential prognostic biomarker and a candidate for further investigation as a therapeutic target. PKLR expression is significantly associated with ARID1A and KRAS mutations.

## 1 Introduction

Cholangiocarcinoma (CCA) is an epithelial cell malignancy originating from the biliary tract, accounting for < 1% of all human malignancies and characterized by high heterogeneity and poor prognosis [1, 2]. The contemporary anatomical classification includes intrahepatic, perihilar, and distal CCA [3]. Intrahepatic CCA is the second most common primary hepatic malignancy [4]. Although surgical techniques and curative liver transplantation have advanced in recent years, the 5‐year survival rate remains low [3]. Perihilar CCA arises in the right and/or left hepatic ducts or at their junction and accounts for approximately 60% of all CCA cases, while distal CCA originates from the epithelium of the common bile duct [1, 5, 6]. Although CCA subtypes are hypothesized to differ in risk factors, pathobiology, clinical presentation, management, and prognosis, large‐scale studies defining these differences are limited [7]. CCA is a rare group of primary neoplasms, yet its incidence is steadily increasing worldwide [6]. Due to genetic heterogeneity, a highly desmoplastic nature, a rich tumor microenvironment, and frequent diagnosis at advanced stages, effective treatments for CCA are limited [3, 4]. Moreover, CCA is often asymptomatic, with nearly 50% of patients presenting with regional lymph node invasion and 25% with distant metastases at initial diagnosis [7]. Therefore, there is an urgent need to develop novel prognostic biomarkers for CCA and to identify potential therapeutic targets.

In recent years, omics approaches have become important for identifying potential therapeutic targets [8, 9]. Based on transcriptome sequencing data from The Cancer Genome Atlas (TCGA) database and Fudan cohorts [4], we identified 10 signatures, FOLH1, PKLR, PTH1R, GPR114, KCNH2, LCN2, MMP10, PORCN, RIPK3, and SRPK3, as potential drug targets for CCA. Among these, decreased expression of pyruvate kinase L/R (PKLR) may be involved in CCA development and represents the most promising therapeutic target. PKLR belongs to the pyruvate kinase (PK) family, which catalyzes the formation of pyruvate and ATP from phosphoenolpyruvate and ADP, playing a crucial role in regulating cell metabolism [10]. In mammals, there are four PK isoforms: PKM1, PKM2, PKL, and PKR [11]. PKM1 is abundantly expressed in high‐energy‐demanding organs such as the brain and muscle [12], while PKM2 is mainly expressed in embryonic cells, adipose tissue, lung, kidney, and cancer cells [12, 13]. PKL is primarily expressed in the liver, kidney, and intestine, and PKR is expressed in red blood cells [11]. To date, targeting PKM1/2, encoded by the PKM gene, has been studied in various malignancies [14, 15, 16]; however, whether PKLR can serve as a therapeutic target for CCA remains unclear.

PKLR gives rise to two isoforms, PKL and PKR [11], and is also involved in maintaining levels of the primary endogenous antioxidant glutathione, regulating lipid metabolism, and modulating mitochondrial activity [17]. Here, we utilized transcriptomic data from CCA patients who underwent surgical resection from the TCGA and Fudan databases, along with three drug databases. We found that PKLR is lowly expressed in CCA tumor tissues, and low PKLR expression is significantly associated with poor prognosis in CCA. Moreover, low PKLR level is an independent risk factor for CCA. PKLR may serve as both a prognostic indicator and a potential therapeutic target for CCA.

## 2 Methods

### 2.1 Study Design and Patients

#### 2.1.1 TCGA Cohort

In this retrospective clinical study, we obtained 36 CCA samples from the TCGA database. Transcriptomic data and clinical files were downloaded from UCSC Xena (https://xenabrowser.net/), and whole‐exome sequencing data were acquired from GDAC Firehose (https://gdac.broadinstitute.org/). UCSC Xena and Firehose provide well‐annotated data from the National Cancer Institute GDC Data Portal.

#### 2.1.2 Fudan Cohort

Chinese hepatobiliary specialist Professor Fan Jia and his team published a study reporting multiomics features of CCA [4]. Transcriptomic, whole‐exome sequencing, and clinical data were obtained from the Supporting Information of this study. A total of 255 samples were collected from Zhongshan Hospital of Fudan University in China, hence termed the Fudan cohort.

All specimens from the TCGA database and Fudan cohort met the following criteria: (a) primary CCA; (b) no other primary cancers; (c) availability of key clinical characteristics; (d) exclusion of patients who died from other causes; and (e) patients who underwent only surgical resection.

After screening, 36 specimens from TCGA and 255 from the Fudan cohort were selected.

A detailed description of the additional CCA cohorts downloaded from the Gene Expression Omnibus (GEO) database is provided in Data S1.

### 2.2 Drug‐Target Mapping

Drug targets with FDA‐approved drugs or candidate drugs under clinical trial for CCA were searched using the DrugBank online database (https://go.drugbank.com/) [18], Selleck website (https://www.selleck.cn/), and MedChemExpress (MCE) website (https://www.medchemexpress.cn/). Signatures were first screened based on expression level and overall survival. Those considered potential drug targets were queried in the aforementioned databases. Only signatures with FDA‐approved targeted drugs, or drugs in clinical or preclinical trials deemed effective, were considered potential therapeutic targets for CCA.

### 2.3 Cell Culture and Cell Lines

The human CCA cell lines Hucc‐T1 (JCRB no. JCRB0425) and RBE (1101HUM‐PUMC000675) were obtained from Japanese Collection of Research Bioresources Cell Bank and Chinese Biomedical Experimental Cell Resource Bank, respectively. All passed tests of cell line quality control methods (e.g., morphology, isoenzymes, and mycoplasma). Cells were cultured in RPMI1640 medium (Gibco, USA) with 10% fetal bovine serum (Gibco, USA). Cells were maintained in a humidified incubator at 37°C with 5% CO_2_. All experiments were performed with cells in the logarithmic growth phase.

### 2.4 Cell Transfection and Small Interfering RNA (siRNA)

To knock down PKLR expression, a specific siRNA targeting PKLR (si‐PKLR) and a negative control siRNA (si‐Cont) were designed and synthesized by GenePharma (Shanghai, China). The si‐PKLR sequences were as follows: sense: 5′‐GAAAGUCUGUGUCCAAUUAUG‐3′, antisense: 5′‐UAAUUGGACACAGACUUUCAG‐3′. Transfection was performed using Lipofectamine 2000 (Invitrogen, USA) according to the manufacturer's protocol. Briefly, cells were seeded into 6‐well plates at a density of 1.5–2.0 × 10^5^ cells/well and cultured overnight to reach 50%–70% confluence. The siRNA–lipid complexes (final siRNA concentration: 50 nM) were added to the cells. After 72 h of transfection, cells were harvested for quantitative real‐time PCR (qRT‐PCR) to confirm knockdown efficiency, or subjected to subsequent functional assays.

### 2.5 qRT‐PCR

Total RNA was extracted from transfected cells using TRIzol reagent (Invitrogen, USA) according to the manufacturer's instructions. First‐strand cDNA was synthesized from 1 μg of total RNA using a PrimeScript RT Reagent Kit (Takara, Japan). qRT‐PCR was performed on an ABI 7500 Real‐Time PCR System using SYBR Green Premix Ex Taq (Takara, Japan). The primer sequences used were as follows: PKLR forward: 5′‐TCAAGGCCGGGATGAACATTG‐3′, reverse: 5′‐CTGAGTGGGGAACCTGCAAAG‐3′; GAPDH (internal control) forward: 5′‐TGGCACCGTCAAGGCTGAGAA‐3′, reverse: 5′‐TGGTGAAGACGCCAGTGGACTC‐3′. Relative gene expression was calculated using the 2^−ΔΔCt^ method.

### 2.6 Cell Proliferation Assay (CCK‐8)

Cell proliferation was assessed using the Cell Counting Kit‐8 (CCK‐8; Dojindo, Japan). At 72 h posttransfection, Hucc‐T1 and RBE cells were trypsinized, counted, and seeded into 96‐well plates at a density of 2000 cells per well (in 100 μL RPMI1640 medium) with three replicate wells per group. After attachment, 10 μL of CCK‐8 reagent was added to each well at indicated time points as follows: 24 h. The plates were incubated for an additional 2 h at 37°C, and the absorbance at 450 nm was measured using a microplate reader (BioRAD, USA). The proliferation curves were plotted based on the mean absorbance values over time.

### 2.7 Cell Migration Assay (Transwell)

Cell migration ability was evaluated using 24‐well Transwell chambers with 8.0 μm pore size polycarbonate membranes (Corning, USA). At 72 h posttransfection, cells were serum‐starved for 6 h, then detached and resuspended in serum‐free medium at a density of 1 × 10^5^ cells/mL. A total of 200 μL of cell suspension was added to the upper chamber, while the lower chamber was filled with 600 μL of complete medium containing 10% FBS as a chemoattractant. After incubation for 24 h at 37°C, the nonmigrated cells on the upper surface of the membrane were gently removed with a cotton swab. The migrated cells on the lower surface were fixed with 4% paraformaldehyde for 15 min and stained with 0.1% crystal violet for 20 min at room temperature. Stained cells were imaged and counted in five randomly selected fields per well under an inverted microscope (Olympus, Japan). The average number of migrated cells per field was calculated for each group.

### 2.8 Statistical Analysis

IBM SPSS Statistics 25.0 and R software 4.1.2 were used for statistical analysis. After data cleansing, including removal of duplicates and meaningless features, the Limma package was used to analyze differential gene expression between tumor and adjacent normal tissues in TCGA patients. *p*‐Values were adjusted using the Benjamini–Hochberg method, with adjusted *p* ≤ 0.05 considered statistically significant.

Standard statistical tests were used to analyze clinical data. Kaplan–Meier analysis assessed the relationship between gene expression and overall survival, with the surv_cutpoint function in the survminer package determining the optimal cut‐off value (Table S1). Log‐rank tests evaluated *p*‐values. Fisher's exact test was used for categorical vs. categorical variables, Kruskal–Wallis test for categorical vs. continuous variables, and Pearson linear regression for continuous vs. continuous variables. Cox proportional hazards regression models were used for univariate and multivariate analyses. Hazard ratios (HRs) and 95% confidence intervals (CIs) were calculated.

A waterfall plot depicting gene mutations and clinical features was generated using the maftools package [19]. A nomogram and calibration curve were developed based on multivariate analysis results using the rms package. Nomogram performance was assessed using the concordance index (C‐index) [20]. A *p* ≤ 0.05 was considered statistically significant (*p ≤ 0.05; **p ≤ 0.01; ***p ≤ 0.001).

### 2.9 Further Materials and Methods

The other detailed materials and methods were provided in the Supporting Information of this manuscript.

## 3 Results

### 3.1 TCGA and Fudan Databases Reveal 10 Potential Therapeutic Targets for CCA

**FIGURE 1:**
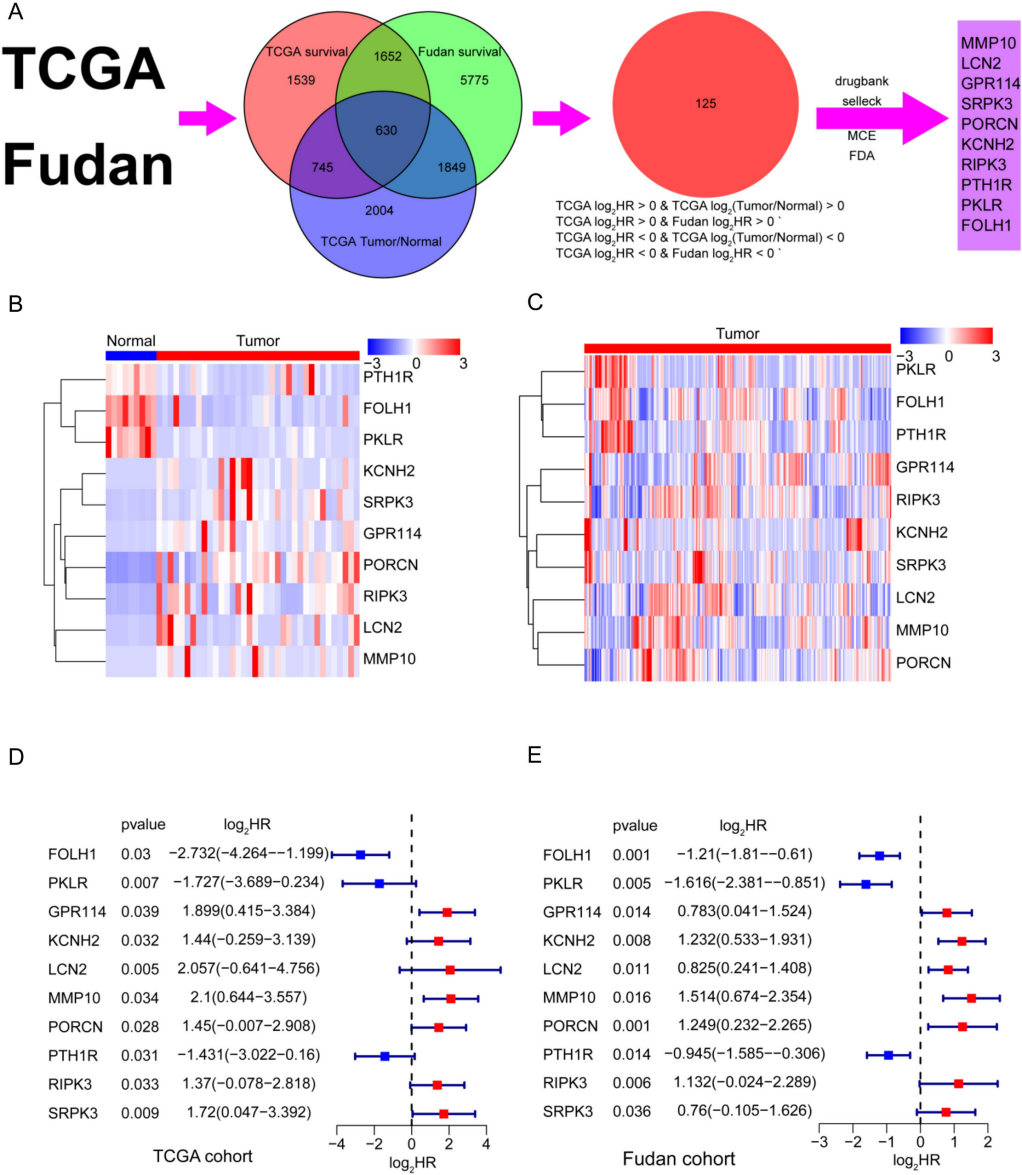
TCGA and Fudan cohorts reveal potential therapeutic targets for CCA. (A) Flow diagram of the screening process for the 10 potential therapeutic targets from TCGA and Fudan cohorts. (B) Heatmap of the expression of 10 potential therapeutic targets in the TCGA cohort. (C) Heatmap of the expression of 10 potential therapeutic targets in the Fudan cohort. (D) Forest plot showing the relationship between CCA patient overall survival and the expression of 10 potential therapeutic targets in the TCGA cohort. (E) Forest plot showing the relationship between CCA patient overall survival and the expression of 10 potential therapeutic targets in the Fudan cohort.

Omics approaches provide a comprehensive framework for delineating tumor landscapes, facilitating precision diagnosis and treatment, and aiding in the discovery of novel biomarkers [21]. Initially, 36 and 255 human CCA specimens were obtained from the TCGA database and the Fudan cohort [4], respectively. As shown in Figure 1A, survival and expression analyses identified 10 signatures that are targets of FDA‐approved drugs or candidate drugs in clinical/preclinical trials (DrugBank, MCE, Selleck). Among these, FOLH1, PKLR, and PTH1R were lowly expressed in CCA tumors compared to adjacent normal tissues, while GPR114, KCNH2, LCN2, MMP10, PORCN, RIPK3, and SRPK3 were highly expressed (Figure 1B,C). We further validated our findings in additional CCA cohorts from the GEO database (see Data S1). Consistently, the same results were obtained from the GSE107943 (non‐CCA: 27, CCA: 30) dataset (Figure S1A). Based on the GSE26566 (adjacent normal: 65, CCA: 104) dataset, FOLH1 and PKLR were lowly expressed in CCA tumors compared to adjacent normal tissues, while GPR114, LCN2, MMP10, and RIPK3 were highly expressed (Figure S1B,C).

Consistently, FOLH1, PKLR, and PTH1R expression positively correlated with CCA patient prognosis, whereas GPR114, KCNH2, LCN2, MMP10, PORCN, RIPK3, and SRPK3 expression negatively correlated with prognosis (Figure 1D,E). Previous studies suggest LCN2 and RIPK3 as promising treatment targets and diagnostic markers for CCA [22, 23]. Simultaneously, FOLH1 and PTH1R were reported to have an association with prostate [24, 25], PKLR was reported to have an association with osteosarcoma [17], KCNH2 was reported regulating melanoma cell proliferation and migration [26], MMP10 was reported facilitating lung metastasis in metastatic renal cancer [27], PORCN was reported promoting hepatocellular carcinogenesis [28]. However, the roles of GPR114 and SRPK3 in CCA and other cancer types remain unclear. Thus, exploring their potential as therapeutic targets and/or diagnostic markers holds significant clinical value.

### 3.2 TCGA and Fudan Databases Reveal the Relationship Between 10 Potential Therapeutic Targets and Proliferation Markers

**FIGURE 2:**
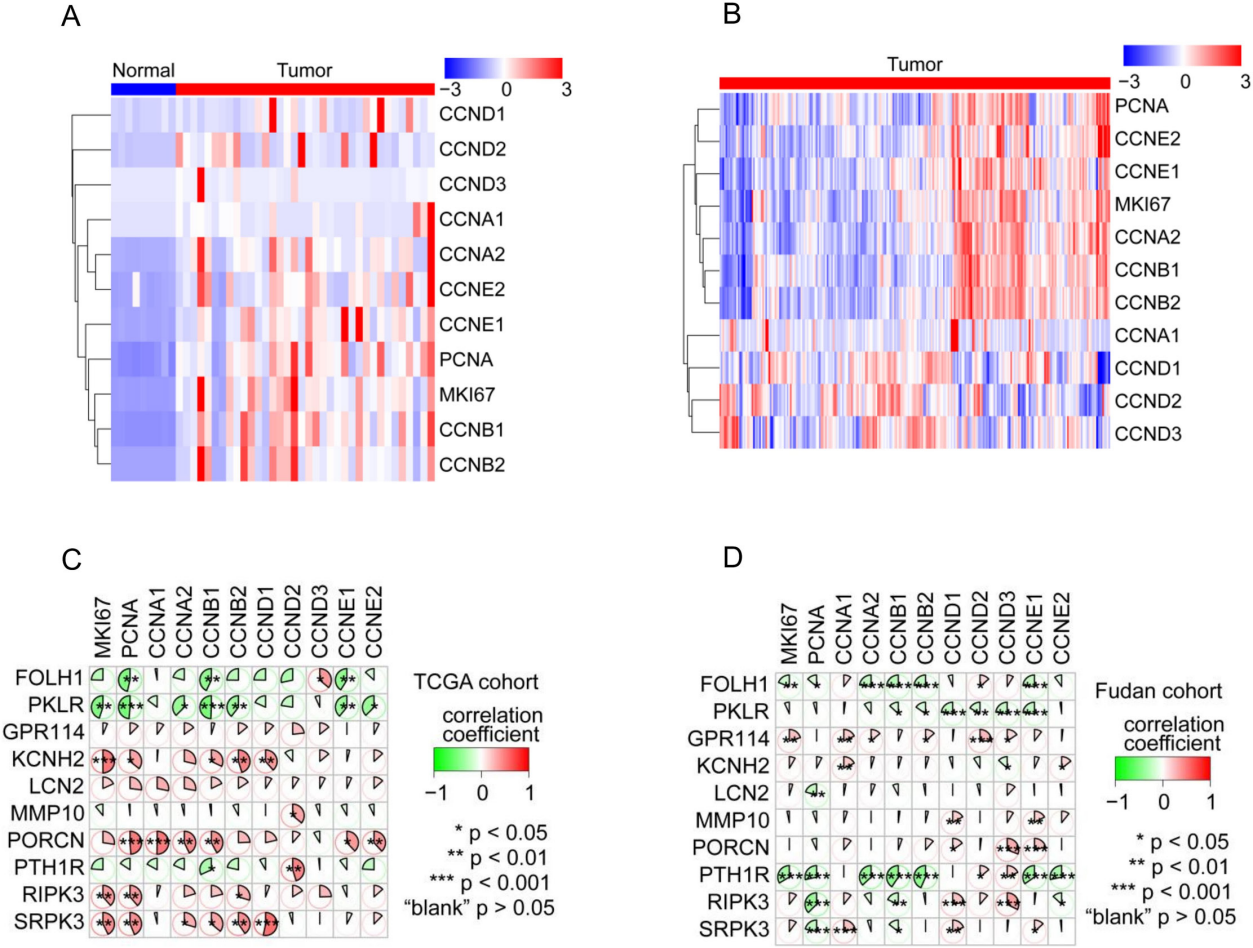
TCGA and Fudan cohorts exhibit the relationship between proliferation markers and the 10 potential prognostic biomarker and therapeutic targets. (A and B) Heatmaps of proliferation marker expression in the TCGA (A) and Fudan (B) cohorts. (C and D) Corrplots showing correlations between proliferation markers and the 10 potential therapeutic targets in the TCGA (C) and Fudan (D) cohorts. Sectors represent correlation coefficients; * indicates statistical significance (*p < 0.05, **p < 0.01, ***p < 0.0001; no symbol indicates *p* ≥ 0.05).

To preliminarily elucidate the role of these 10 signatures in CCA proliferation, we examined their expression alongside general proliferation markers, including MKI67, PCNA, CCNA1, CCNA2, CCNB1, CCNB2, CCND1, CCND2, CCND3, CCNE1, and CCNE2 [29, 30, 31, 32, 33]. Based on TCGA data, these markers were significantly upregulated in CCA tumors compared to normal tissues (Figure 2A), with notable heterogeneity across samples (Figure 2A,B). The three genes, FOLH1, PKLR, PTH1R, which were downregulated in tumors and correlated with better prognosis, acting as potential tumor suppressors, were negatively correlated with most proliferation markers, whereas the seven oncogenes (GPR114, KCNH2, LCN2, MMP10, PORCN, RIPK3, SRPK3) were positively correlated (Figure 2C,D). Among these, PKLR showed a consistent negative correlation with all proliferation markers in both TCGA and Fudan databases, suggesting it may play an important role in inhibiting CCA development.

### 3.3 TCGA and Fudan Databases Reveal the Relationship Between 10 Potential Therapeutic Targets and Metastasis Markers

**FIGURE 3:**
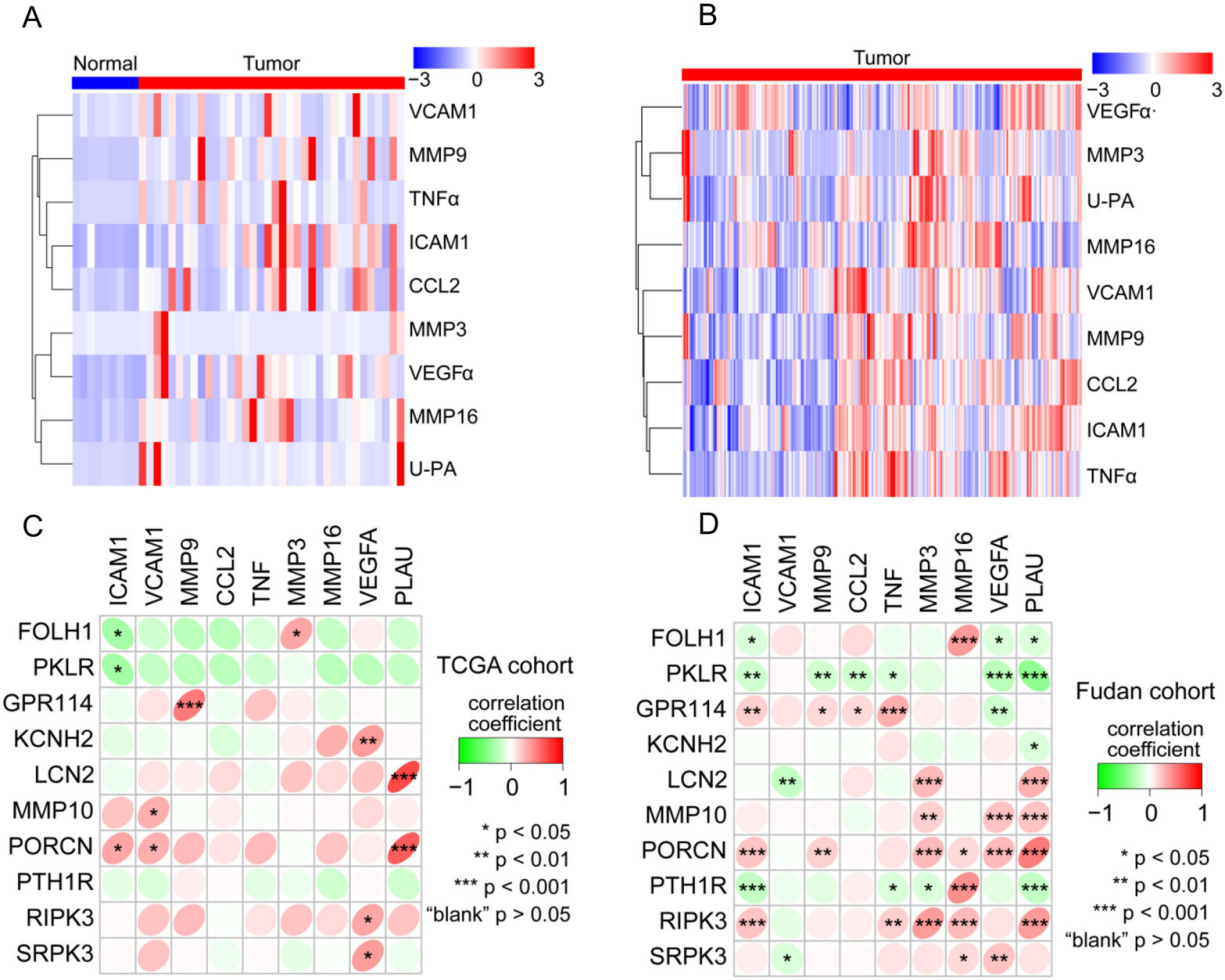
TCGA and Fudan cohorts exhibit the relationship between metastasis markers and the 10 potential prognostic biomarker and therapeutic targets. (A and B) Heatmaps of metastasis marker expression in the TCGA (A) and Fudan (B) cohorts. (C and D) Corrplots showing correlations between metastasis markers and the 10 potential therapeutic targets in the TCGA (C) and Fudan (D) cohorts. Ellipses represent correlation coefficients; * indicates statistical significance (*p < 0.05, **p < 0.01, ***p < 0.0001; no symbol indicates *p* ≥ 0.05).

Metastasis markers, including ICAM1, VCAM1, MMP9, CCL2, TNFA, MMP3, MMP16, VEGFA, and u‐PA [34, 35, 36, 37], were significantly upregulated in tumor tissues compared to normal tissues (Figure 3A), with considerable heterogeneity across samples (Figure 3A,B). Linear regression analysis revealed that the three tumor suppressor genes (FOLH1, PKLR, PTH1R) were negatively correlated with most metastasis markers, while the seven oncogenes (GPR114, KCNH2, LCN2, MMP10, PORCN, RIPK3, SRPK3) were positively correlated (Figure 3C,D). PKLR consistently showed a negative correlation with all general metastasis markers, indicating its potential role in inhibiting CCA metastasis and reinforcing its candidacy as a therapeutic target.

### 3.4 TCGA and Fudan Databases Reveal the Relationship Between 10 Potential Therapeutic Targets and High Frequency Mutated Genes in CCA

**FIGURE 4:**
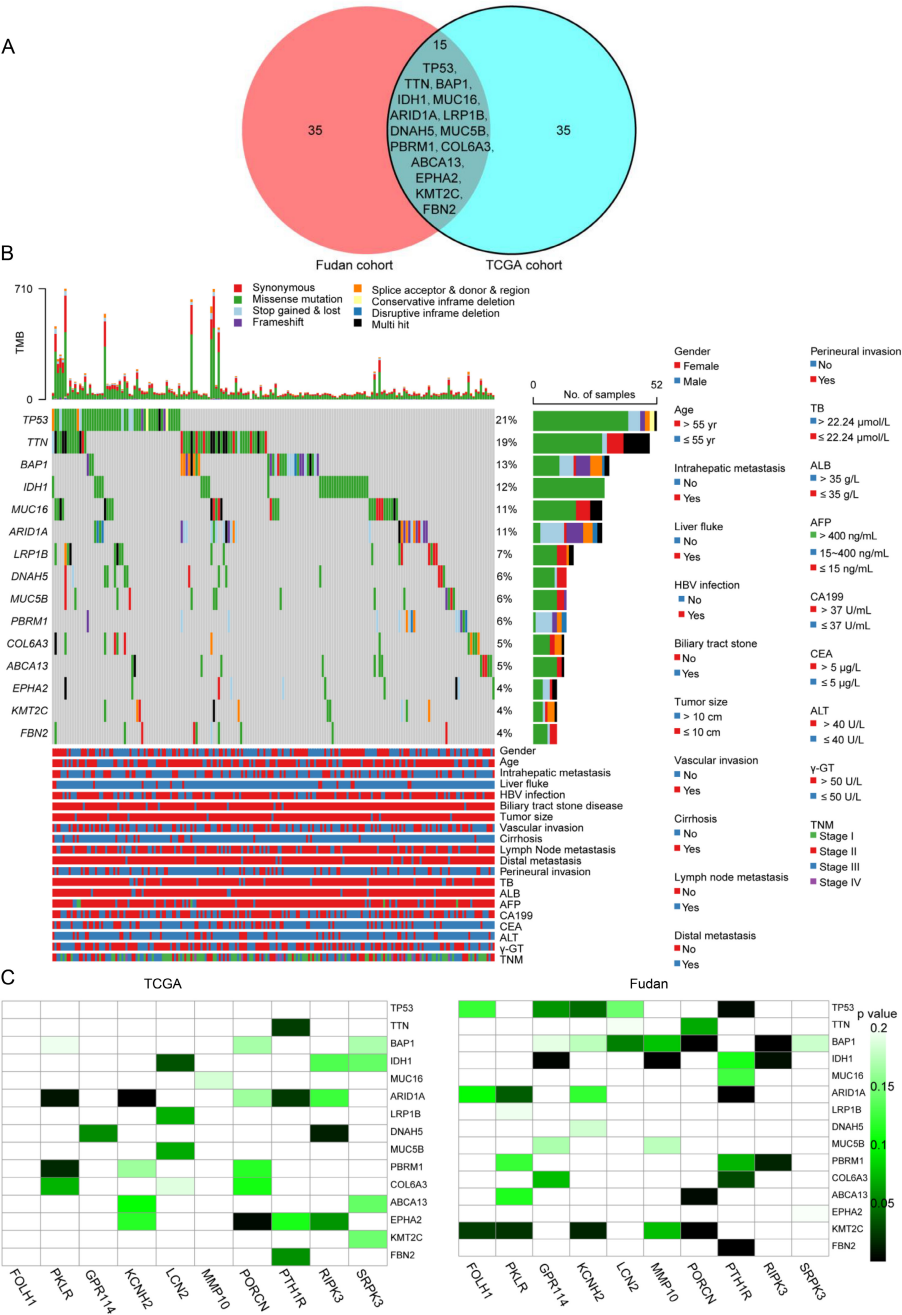
TCGA and Fudan cohorts exhibit the relationship between 10 potential signatures and the most frequently mutated genes in CCA. (A) Venn diagram identifying 15 genes common to the top 50 most frequently mutated genes in both TCGA and Fudan cohorts. (B) Waterfall plot of the 15 most frequently mutated genes and clinical characteristics in the Fudan cohort. (C) Heatmaps showing the relationship between the 15 most frequently mutated genes and the 10 potential therapeutic targets in the TCGA (left) and Fudan (right) cohorts.

Mutated driver genes promote tumorigenesis across various organisms [38]. To explore the relationship between potential signatures and genetic alterations in CCA, we first analyzed the high‐frequency mutated genes in TCGA and Fudan cohorts. The top 50 frequently mutated genes from each database were compared, and 15 genes, including TP53, TTN, BAP1, IDH1, MUC16, ARID1A, LRP1B, DNAH5, MUC5B, PBRM1, COL6A3, ABCA13, EPHA2, KMT2C, and FBN2, were identified as the high‐frequency mutated genes in CCA (Figure 4A). A waterfall plot illustrated the mutation landscape of these 15 genes in the Fudan cohort (Figure 4B). TP53 was the most frequently mutated gene, occurring in 21% of patients (Figure 4B). No potential signature showed a significant relationship with TP53 mutations in the TCGA cohort, whereas KCNH2 and PTH1R were associated with TP53 mutations in the Fudan cohort (Figure 4C). PKLR expression was significantly associated with ARID1A mutation status in both cohorts (Figure 4C).

Due to the small sample size of the TCGA cohort, the list of frequently mutated genes is not exhaustive. KRAS is a key gene with a high‐frequency mutation in CCA [4], we explored the relationship of KRAS mutation and the 10 potential therapeutic targets and proliferation markers. As shown in Figure S2A, PKLR, PTH1R, LCN2, MMP10, PORCN, RIPK3, and SRPK3 were significantly associated with KRAS mutation in the Fudan cohort. Because of the limited mutant KRAS patients in the TCGA cohort (*n* = 2), we could not perform statistical analysis; thus, only the expression levels of the 10 signatures were shown in Figure S2B.

### 3.5 PKLR Is an Independent Protective Factor for Prognosis in CCA

**FIGURE 5:**
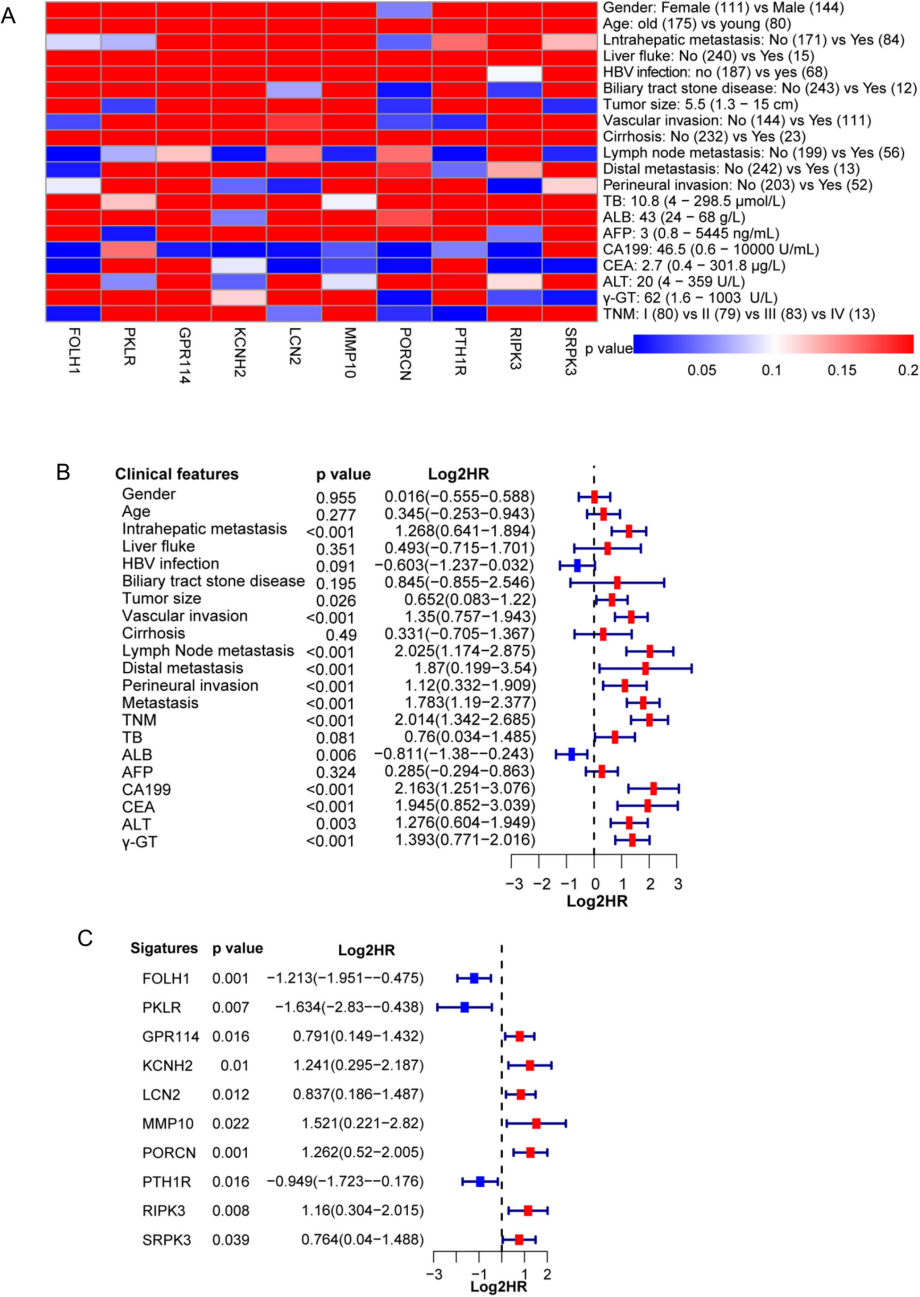
PKLR is suggested as an independent predictor of poor overall survival in patients with CCA. (A) Heatmap showing associations between clinical characteristics and the 10 potential therapeutic targets in the Fudan cohort. *p*‐Values are listed in cells. (B) Forest plot of Kaplan–Meier analysis for overall survival based on clinical features in the Fudan cohort. (C) Forest plot of univariate analyses for the 10 potential therapeutic targets in the Fudan cohort.

To further assess the role of the 10 potential targets in CCA, we examined their relationship with clinical features in the Fudan cohort (*n* = 255). PORCN showed the most significant associations with clinical characteristics and the second highest HR among all biomarkers (Figures 1D,E and 5A), indicating its role as a cancer‐promoting factor and a promising therapeutic target. Consistent with prior findings, PKLR was significantly associated with tumor size (Wilcoxon rank‐sum test, *p* = 0.024), metastasis (intrahepatic, lymph node, and distant metastasis; Fisher's exact test, *p* = 0.014), and AFP level (Wilcoxon rank‐sum test, *p* = 0.008), supporting its role as a tumor suppressor and a viable therapeutic target.

We further analyzed the relationship between clinical characteristics and prognosis in the Fudan cohort. Intrahepatic metastasis, tumor size, vascular invasion, lymph node metastasis, distant metastasis, perineural invasion, TNM stage, ALB, CA199, CEA, ALT, and γ‐GT were significantly associated with CCA prognosis (Figure 5B), highlighting their importance as prognostic indicators and potential therapeutic focuses.

Univariate Cox regression analysis identified the expression levels of the 10 signatures, along with the above clinical features, as factors influencing overall survival (OS) (Figure 5C and Tables 1, 2, S2, and S3). Multivariate analysis based on Fudan cohort confirmed that only PKLR expression, lymph node metastasis, CA199, ALT, and TNM stage were independent risk factors for OS (Tables 3 and S4). Multivariate analysis based on TCGA cohort confirmed that only PKLR expression and tumor status were independent risk factors for OS (Table S3). These results indicate that low PKLR expression is an independent risk factor associated with poor clinical outcomes in CCA patients. Based solely on our computational analysis, PKLR represents a candidate for further investigation as a therapeutic target. The potential use of the FDA‐approved PKLR activator Mitapivat [39] in CCA would require extensive preclinical validation.

**TABLE 1:** Univariate analyses of associations between overall survival and 10 potential signatures in the Fudan cohort (*n* = 255).

Univariable	Patient's numbers	HR	95% CI low	95% CI high	*p*
FOLH1	79Versus. 176	0.431	0.259	0.719	**0.001**
PKLR	33Versus. 222	0.322	0.141	0.738	**0.007**
GPR114	49Versus. 206	1.730	1.109	2.698	**0.016**
KCNH2	210Versus. 45	2.363	1.227	4.553	**0.010**
LCN2	169Versus. 86	1.786	1.138	2.803	**0.012**
MMP10	228Versus. 27	2.870	1.166	7.063	**0.022**
PORCN	30Versus. 225	2.399	1.434	4.013	**0.001**
PTH1R	60Versus. 195	0.518	0.303	0.885	**0.016**
RIPK3	26Versus. 229	2.234	1.235	4.042	**0.008**
SRPK3	36Versus. 219	1.698	1.028	2.804	**0.039**

*Note:* Bold values indicate statistical significance (*p* < 0.05).

**TABLE 2:** Univariate analyses of associations between overall survival and clinical features in the Fudan cohort (*n* = 255).

Univariables^a^		HR	95% CI low	95% CI high	*p*
Gender (male vs. female)	111 vs. 144	0.989	0.665	1.470	0.955
Age (Young vs old)	175 vs. 80	1.271	0.824	1.960	0.278
Intrahepatic metastasis (yes vs. no)	171 vs. 84	2.428	1.634	3.607	**< 0.0001**
Liver fluke (yes vs. no)	240 vs. 15	1.409	0.683	2.906	0.353
HBV (yes vs. no)	187 vs. 68	0.657	0.401	1.074	0.094
Biliary tract stone disease (yes vs. no)	243 vs. 12	1.800	0.731	4.433	0.201
Tumor size (cm)	5.5 (1.3–15)	1.151	1.066	1.241	**< 0.0001**
Vascular invasion (yes vs. no)	144 vs. 111	2.584	1.727	3.866	**< 0.0001**
Cirrhosis (yes vs. no)	232 vs. 23	1.259	0.654	2.425	0.491
Lymph node metastasis (yes vs. no)	199 vs. 56	4.180	2.765	6.318	**< 0.0001**
Distal metastasis (yes vs. no)	242 vs. 13	3.817	1.957	7.446	**< 0.0001**
Perineural invasion (yes vs. no)	203 vs. 52	2.197	1.413	3.416	**< 0.0001**
TB (μmol/L)	10.8 (4–298.5)	1.004	0.996	1.012	0.310
ALB (g/L)	(24–68)	0.966	0.928	1.005	0.088
AFP (ng/mL)	(0.8–5445)	1.000	1.000	1.000	0.990
CA199 (U/mL)	46.5 (0.6–10 000)	1.000	1.000	1.000	**< 0.0001**
CEA (μg/L)	2.7 (0.4–301.8)	1.011	1.007	1.015	**< 0.0001**
ALT (U/L)	20 (4–359)	1.003	1.000	1.006	**0.026**
γ‐GT (U/L)	62 (1.6–1003)	1.002	1.000	1.003	**0.007**
TNM stage (I vs. II vs. III vs. IV)	80 vs. 79 vs. 83 vs. 13	2.236	1.786	2.799	**< 0.0001**

*Note:* Bold values indicate statistical significance (*p* < 0.05).

^a^ For continuous variables, mean, minimum, maximum values were calculated. For categorical data, patient number were calculated.

**TABLE 3:** Multivariate analyses of associations between overall survival and various risk factors in the Fudan cohort (*n* = 255).

Multivariables^a^		HR	95% CI low	95% CI high	*p*
PKLR (high vs. low)	33 vs. 222	0.313	0.132	0.742	**0.008**
Lymph node metastasis (yes vs. no)	199 vs. 56	2.160	1.320	3.533	**0.002**
CA199 (U/mL)	46.5 (0.6–10 000)	1.000	1.000	1.000	**< 0.0001**
ALT (U/L)	20 (4–359)	1.004	1.001	1.007	**0.015**
TNM stage (I vs. II vs. III vs. IV)	80 vs. 79 vs. 83 vs. 13	1.685	1.282	2.213	**< 0.0001**

*Note:* Bold values indicate statistical significance (*p* < 0.05).

^a^ For continuous variables, mean, minimum, maximum values were calculated. For categorical data, patient number were calculated.

Contrary to the established role of PKLR as an oncogene in various extrahepatic malignancies, including osteosarcoma, prostate cancer, pancreatic cancer, gallbladder cancer, breast cancer, and colorectal cancer [17, 40, 41, 42, 43], our transcriptome‐wide analysis using TCGA database revealed a more complex expression pattern. Comparative analysis of tumor tissues versus their matched adjacent normal tissues demonstrated that PKLR was significantly upregulated in six tumor types (Figure S3A), while its expression remained unchanged in 11 others (Figure S3B). The PKLR gene encodes two pyruvate kinase isoforms, PKL and PKR, with PKL expression being largely restricted to the liver, kidney, and intestine [11]. Intriguingly, PKLR expression was significantly downregulated in six additional tumor types, all of which originate from these PKL‐specific organs (Figure S4A,B). This dichotomous expression profile suggests that the two isoforms, despite being encoded by the same gene, may exert opposing functions in tumorigenesis.

### 3.6 PKLR Plays an Important Role in Evaluating the Prognosis of Patients With CCA

To investigate the prognostic role of PKLR, 249 CCA patients (Fudan cohort, six cases missing mutation data) and 36 CCA patients were analyzed. Due to all patients in the Fudan cohort were iCCA, while patients in the TCGA cohort included dCCA (*n* = 2, 5.56%), iCCA (*n* = 30, 83.33%), and hCCA (*n* = 4, 11.11%), subtype analysis of the TCGA cohort was performed. As for PKLR expression, there was no difference between iCCA and hCCA (Figure S5A). Consistently, iCCA patients with low expression of PKLR had shorter OS than iCCA patients with high expression of PKLR (Figure S5B).

**FIGURE 6:**
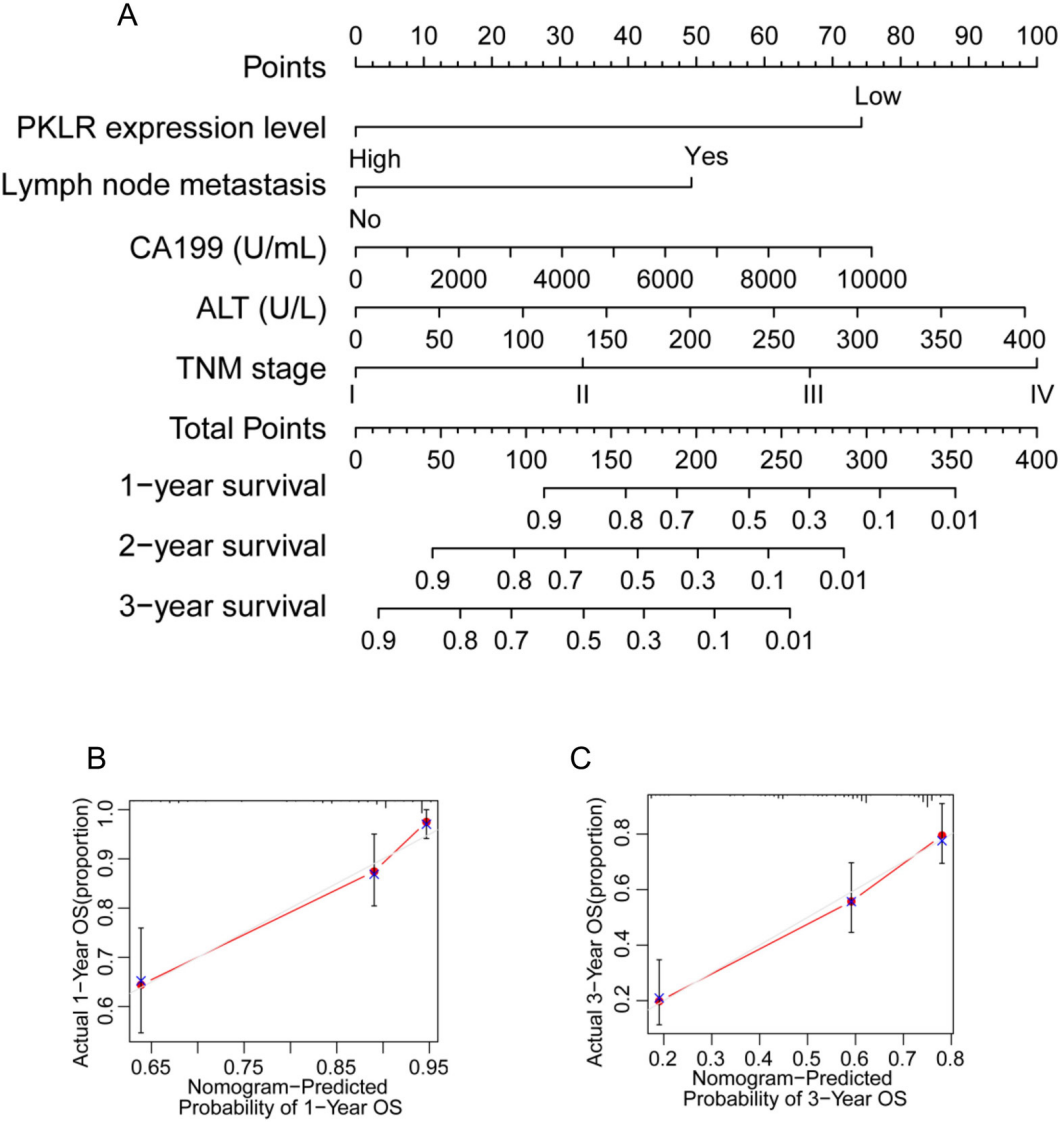
Cholangiocarcinoma survival nomogram. (A) The nomogram assigns points for each variable; total points correspond to predicted 1‐, 2‐, or 3‐year survival probabilities. (B and C) Calibration curves for 1‐year (B) and 3‐year (C) survival in the Fudan cohort. The *x*‐axis represents nomogram‐predicted probability, and the *y*‐axis represents actual overall survival. The red line indicates predictions, and the gray line represents ideal performance.

Furthermore, a prognostic nomogram integrating PKLR and other significant independent clinical features was constructed (Figure 6A). The C‐index for OS prediction was 0.764. Low PKLR expression contributed over 72 points to the total score, exceeding the contributions of lymph node metastasis and TNM stages I–III. Only CA199 > 10 000 U/mL, ALT > 300 U/L, and TNM stage IV contributed higher points, underscoring the prognostic significance of PKLR. Calibration plots for 1‐ and 3‐year survival showed excellent agreement between nomogram predictions and actual observations (Figure 6B,C).

### 3.7 Silencing PKLR Expression Promotes Growth and Metastasis of CCA In Vitro

**FIGURE 7:**
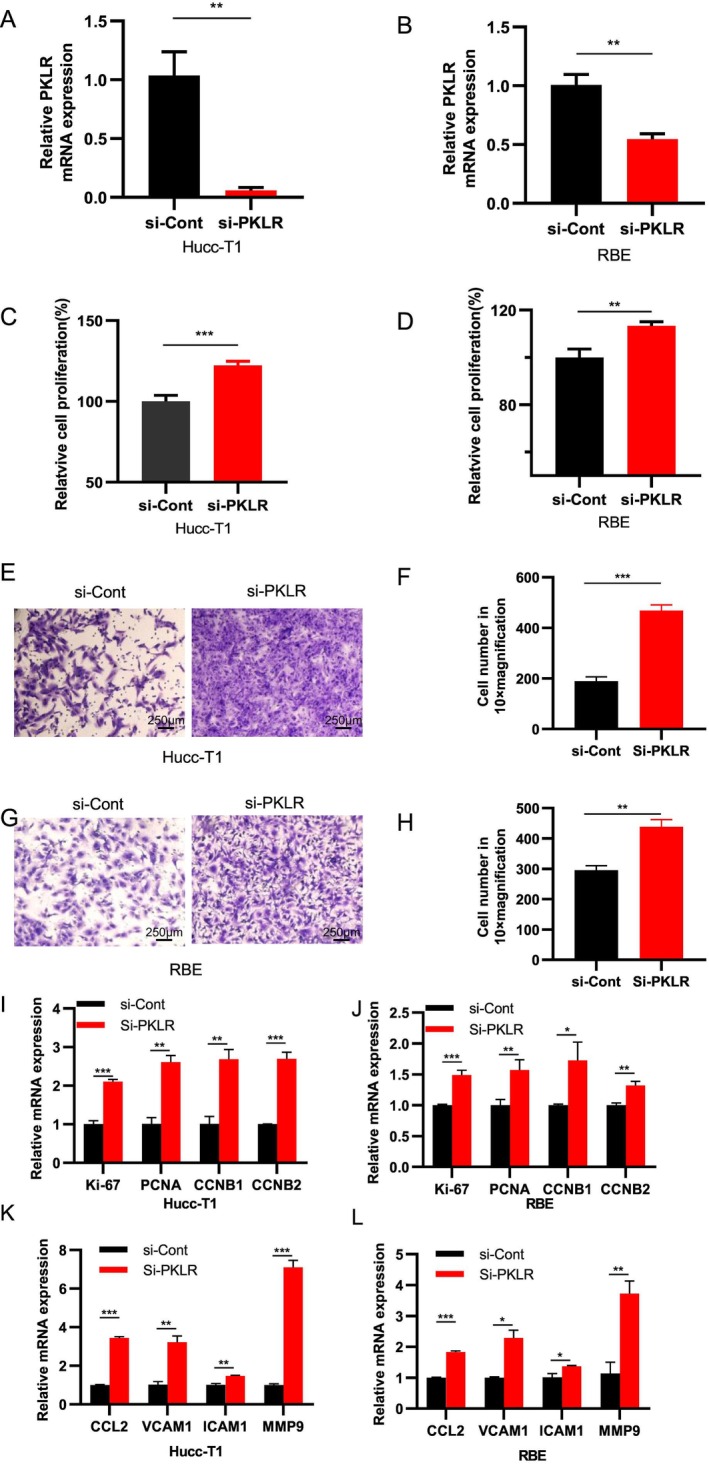
Inhibiting PKLR mRNA expression promoting CCA cell proliferation and metastasis. The si‐PKLR can significantly inhibit PKLR expression in both Hucc‐T1 (A) and RBE (B) cell lines. Si‐PKLR can significantly promote CCA proliferation after 72 h transfection in both Hucc‐T1 (C) and RBE (D) cell lines. Si‐PKLR can significantly promote CCA metastasis after 72 h transfection in both Hucc‐T1 (E and F) and RBE (G and H) cell lines. Si‐PKLR can significantly promote proliferation‐related markers expression in both Hucc‐T1 (I) and RBE (J) cell lines. Si‐PKLR can significantly promote proliferation‐related markers expression in both Hucc‐T1 (K) and RBE (L) cell lines. All statistics were based on at least three repetitions. * was < 0.05, ** was < 0.01, *** was < 0.001. Error bar was calculated based on SEM.

To further validate the protective roles of PKLR in CCA, we evaluated the effects of PKLR on cell proliferation and migration in the CCA cell lines Hucc‐T1 and RBE. The results demonstrated that silencing of PKLR significantly reduced PKLR mRNA expression (Figure 7A,B) and promoted proliferation and migration of CCA cells (Figure 7C–H). To examine the relationship between PKLR expression and proliferation‐ and metastasis‐related markers, we detected the expression of these markers following PKLR silencing. Consistently, the expression levels of all proliferation‐ and metastasis‐related markers were significantly higher in si‐PKLR CCA cells than in si‐Cont CCA cells (Figure 7I–L). Taken together, PKLR acts as a tumor suppressor in CCA.

## 4 Discussion

CCA, a malignancy originating from the biliary tract, is characterized by high heterogeneity and poor prognosis [1]. Unfortunately, CCA mortality has increased globally in recent decades [2]. Despite advances in identifying oncogenic drivers and developing targeted therapies, CCA remains a fatal and treatment‐refractory malignancy [1]. Anatomically, CCA is classified into intrahepatic, perihilar, and distal subtypes [3]. Although these subtypes may differ in risk factors, they all arise from biliary epithelial cells and differences are limited [7, 44]. In this study, we leveraged TCGA data and a published dataset [4] to identify potential therapeutic targets and diagnostic biomarkers for CCA.

Through multiomics analysis, we identified PKLR as the most promising candidate for a prognostic biomarker and a potential therapeutic target for CCA. PKLR is a member of the pyruvate kinase family, which plays a critical role in glycolysis [45], and PKLR has FDA‐approved targeted drugs, Mitapivat. However, the use of Mitapivat on CCA would require extensive preclinical validation.

Glycolysis converts glucose to pyruvate, with pyruvate kinase serving as the third rate‐limiting enzyme, catalyzing the conversion of phosphoenolpyruvate and ADP to pyruvate and ATP [45, 46]. Glycolysis is not only essential for glucose metabolism but also implicated in tumor progression [46, 47]. Dysregulation of pyruvate kinase has been linked to various cancers, including colorectal, ovarian, and breast cancer [48, 49, 50].

Mammals express four pyruvate kinase isoforms: PKM1, PKM2, PKL, and PKR [11]. PKLR encodes PKL and PKR, expressed primarily in the liver and red blood cells, respectively [12, 13]. The role of PKLR as a therapeutic target or diagnostic biomarker in CCA remains unexplored. In this study, we demonstrated that low PKLR expression is associated with increased clinical risk in CCA, including larger tumor size and metastasis. Nomograms have proven effective in predicting cancer prognosis [20]; thus, we developed a nomogram incorporating PKLR expression. Low PKLR expression contributed substantially to the total risk score, indicating that CCA patients with low PKLR expression have poorer prognosis and shorter survival, and suggesting that PKLR activation may mitigate risk. These findings suggest that PKLR activation could be explored as a potential strategy to improve survival in this patient group.

Surprisingly, high PKLR expression in extrahepatic organs has been reported to promote tumor progression in osteosarcoma, prostate cancer, pancreatic cancer, gallbladder cancer, breast cancer, and colorectal cancer [17, 40, 41, 42, 43]. However, in both the Fudan (*n* = 255) and TCGA (*n* = 36) cohorts, PKLR expression was negatively correlated with proliferation and metastasis markers, and low PKLR expression was associated with poor prognosis. In addition, PKLR expression was significantly lower in CCA tumor tissues than adjacent normal tissues. After a transcriptome‐wide analysis, we speculate that the protective role of PKLR in CCA may be mediated by the PKL isoform, which is predominantly expressed in the liver, kidney, and intestine [12, 13], a hypothesis warranting further investigation.

Given the high therapeutic resistance and treatment challenges in CCA, novel targeted therapies are urgently needed [51]. Whole‐exome sequencing has identified frequently mutated genes in CCA [4, 52]. We examined the relationship between PKLR expression and these mutations and found that PKLR expression differed significantly only between ARID1A‐mutated and wild‐type cases. ARID1A encodes a key subunit of the SWI/SNF complex, involved in tumorigenesis via β‐catenin and c‐MYC/PARP1 pathways [53, 54]. ARID1A mutations define a distinct tumor phenotype [55], and their association with PKLR expression suggests a role for PKLR in ARID1A‐related signaling.

In this study, we further performed in vitro functional assays to validate the tumor‐suppressive role of PKLR in CCA. Silencing of PKLR in Hucc‐T1 and RBE cells significantly promoted cell proliferation and migration, accompanied by upregulation of proliferation‐ and metastasis‐related markers. These experimental results are fully consistent with our bioinformatics findings that low PKLR expression correlates with aggressive clinicopathological features and poor prognosis in CCA patients. Collectively, our data demonstrate that PKLR acts as a bona fide tumor suppressor in CCA, and its downregulation may contribute to disease progression. These findings provide a strong rationale for further exploring PKLR as a prognostic biomarker and a potential target for therapeutic activation in CCA.

This is an initial exploratory study for CCA, and our findings are hypothesis‐generating and that therapeutic claims are speculative without enough experimental validation. In summary, low PKLR expression identifies a subgroup of CCA patients with aggressive disease and is closely correlated with tumor size and metastasis. PKLR serves as a prognostic biomarker for predicting OS and a potential therapeutic target for CCA. Patients with low PKLR expression are predicted to have poor survival. PKLR appears closely linked to ARID1A and KRAS mutations. Silencing PKLR expression promotes growth and metastasis of CCA in vitro.

However, there are still several significant limitations: (1) The primary limitation of this study is the lack of sufficient in vitro or in vivo experimental validation (cell proliferation, invasion, or apoptosis assays following PKLR overexpression are necessary) to support the correlational findings. (2) The conclusions regarding PKLR as a therapeutic target are speculative without mechanistic or functional studies. (3) Our sample sizes, particularly the TCGA cohort (*n* = 36), are limited, and the heterogeneity of CCA subtypes was not fully accounted for, and the list of frequently mutated genes was not exhaustive due to data availability. Therefore, all findings should be considered hypothesis‐generating.

Based on these limited findings, our future aims are to: (1) elucidate the functions of PKL and PKR in CCA and determine whether PKL plays a predominant role; (2) clarify the mechanistic role of PKLR in CCA, particularly in ARID1A‐mutated cases; and (3) validate PKLR as an effective therapeutic target for CCA treatment.

## Author Contributions

**Biyan Gong:** methodology, funding acquisition, investigation, data curation, writing – original draft. **Cailing Li:** investigation. **Tubing Xu:** formal analysis, investigation. **Yuandeng Luo:** writing – review and editing, writing – original draft, conceptualization, methodology, validation, investigation, visualization, software, formal analysis, data curation, supervision, resources, project administration. **Dong Zhang:** investigation. **Tingjun Li:** data curation, supervision, resources, investigation, project administration. **Xuejun Xu:** investigation, writing – original draft, visualization, validation. **Li Yang:** investigation.

## Funding

This work was supported by the Natural Science Foundation of Chongqing General Project for Biyan Gong (CSTB2023NSCQ‐MSX1066).

## Conflicts of Interest

The authors declare no conflicts of interest.

## Supporting information


**Data S1:** cnr270649‐sup‐0001‐supinfo.docx.
**Table S1:** Best cutoffs of 10 signatures using surv_cutpoint function in the R survminer package.
**Table S2:** Univariate analyses of associations between overall survival and 10 potential signatures in the TCGA cohort (*n* = 36).
**Table S3:** Univariate analyses of associations between overall survival and clinical features in the TCGA cohort (*n* = 36).
**Table S4:** Multivariate analyses of associations between overall survival and various risk factors in the TCGA cohort (*n* = 36).

**Figure S1:** The expression levels of the 10 potential prognostic biomarker and therapeutic targets between nontumor tissues and CCA tumor tissues in GSE GSE26566 and GSE107943 cohorts.
**Figure S2:** The relationship of the 10 potential prognostic biomarker and therapeutic targets between mutated KRAS.
**Figure S3:** Differential expression of PKLR across 17 malignancies.
**Figure S4:** PKLR expression in malignancies originating from PKL‐specific organs.
**Figure S5:** The expression level of PKLR among three subtypes of CCA in TCGA cohort.

## Data Availability

Data sharing not applicable to this article as no datasets were generated or analyzed during the current study.
