# Supplementary material for: Therapeutic and Predicted Significance of PKLR in Patients With Cholangiocarcinoma
Source: Cancer Rep (Hoboken). 2026 Aug 14;9(8):e70649. doi: 10.1002/cnr2.70649 (PMC13475273; doi:10.1002/cnr2.70649)
Supplement: Supplementary file 1 [file CNR2-9-e70649-s001.pdf]

Figure.S1

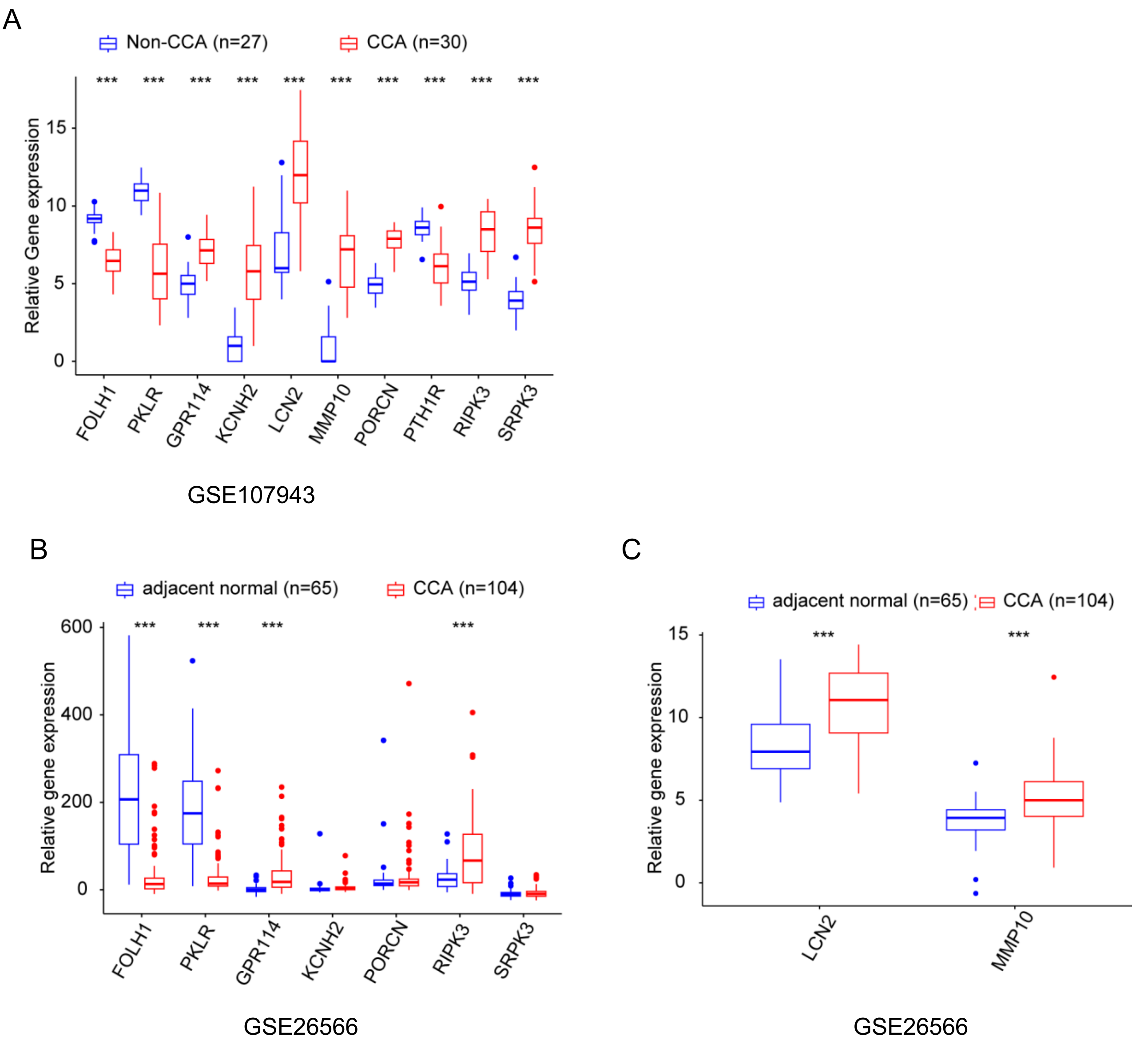
A
Non-CCA (n=27)
CCA (n=30)
***
Relative Gene expression
0
5
10
15
FOLH1
PKLR
GPR114
KCNH2
LCN2
MMP10
PORCN
PTH1R
RIPK3
SRPK3
GSE107943
B
adjacent normal (n=65)
CCA (n=104)
Relative gene expression
0
200
400
600
FOLH1
PKLR
GPR114
KCNH2
PORCN
RIPK3
SRPK3
GSE26566
C
adjacent normal (n=65)
CCA (n=104)
Relative gene expression
0
5
10
15
LCN2
MMP10
GSE26566

Figure.S2

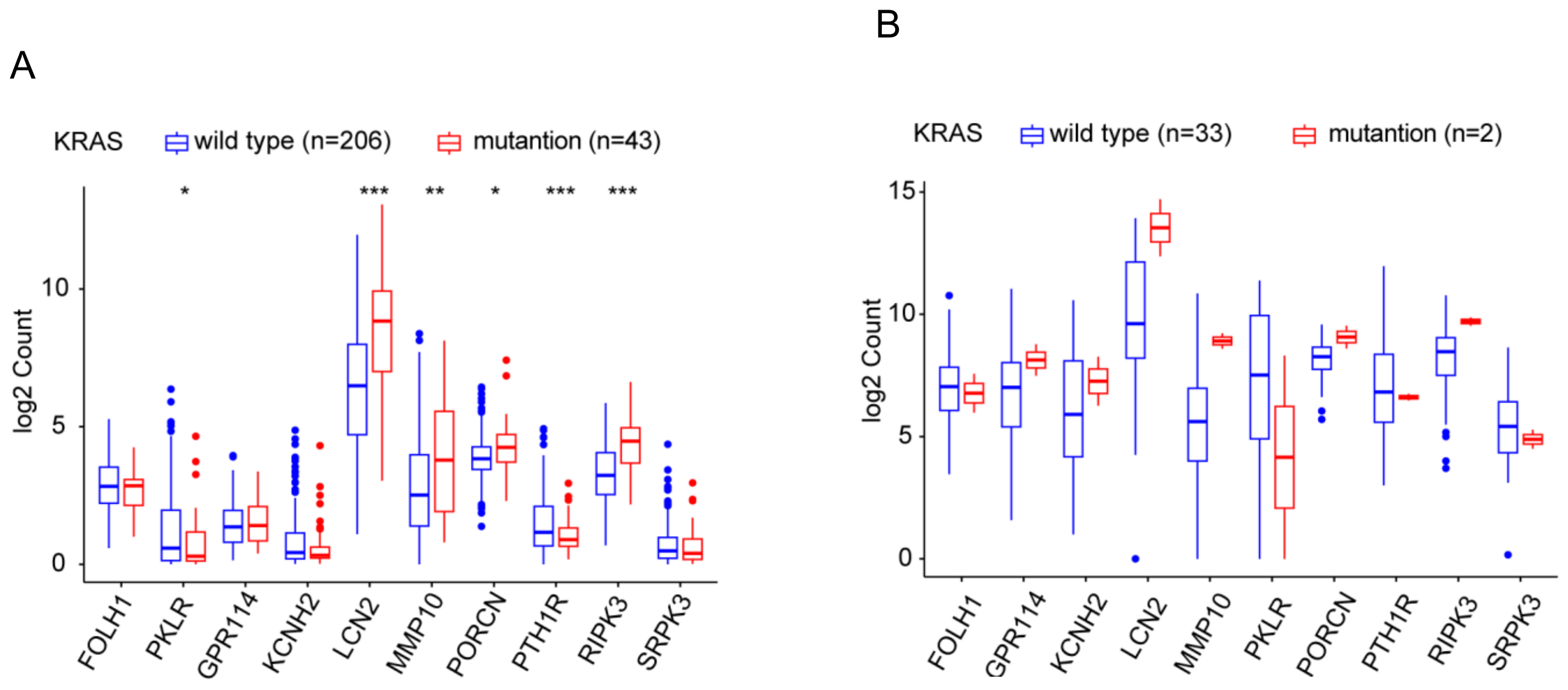
A
KRAS
wild type (n=206)
mutantion (n=43)
log2 Count
0
5
10
*
***
**
*
***
***
FOLH1
PKLR
GPR114
KCNH2
LCN2
MMP10
PORCN
PTH1R
RIPK3
SRPK3
B
KRAS
wild type (n=33)
mutantion (n=2)
log2 Count
0
5
10
15
FOLH1
GPR114
KCNH2
LCN2
MMP10
PKLR
PORCN
PTH1R
RIPK3
SRPK3

Figure.S3

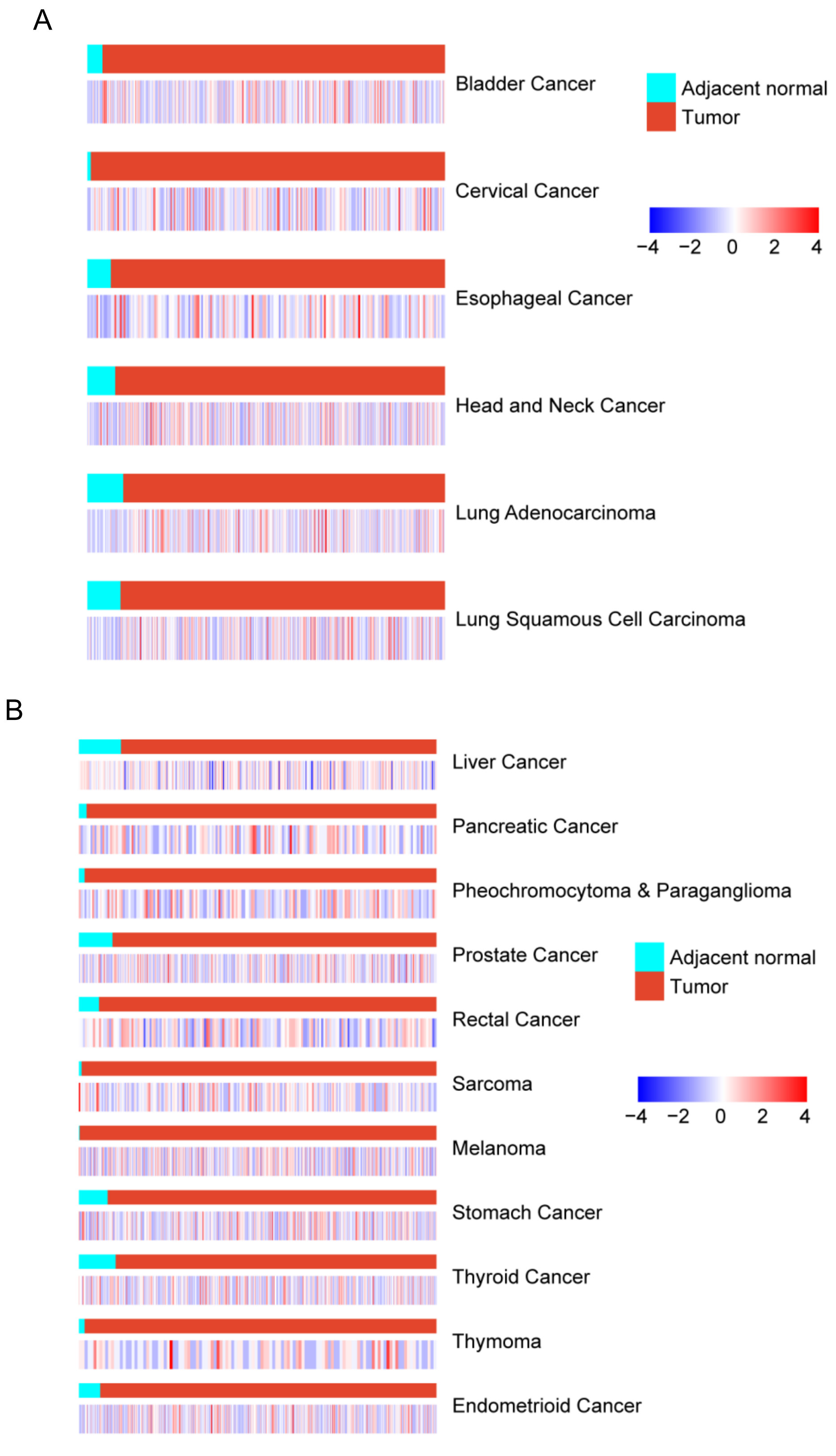
A
Bladder Cancer
Cervical Cancer
Esophageal Cancer
Head and Neck Cancer
Lung Adenocarcinoma
Lung Squamous Cell Carcinoma
Adjacent normal
Tumor
−4 −2 0 2 4
B
Liver Cancer
Pancreatic Cancer
Pheochromocytoma & Paraganglioma
Prostate Cancer
Rectal Cancer
Sarcoma
Melanoma
Stomach Cancer
Thyroid Cancer
Thymoma
Endometrioid Cancer
Adjacent normal
Tumor
−4 −2 0 2 4

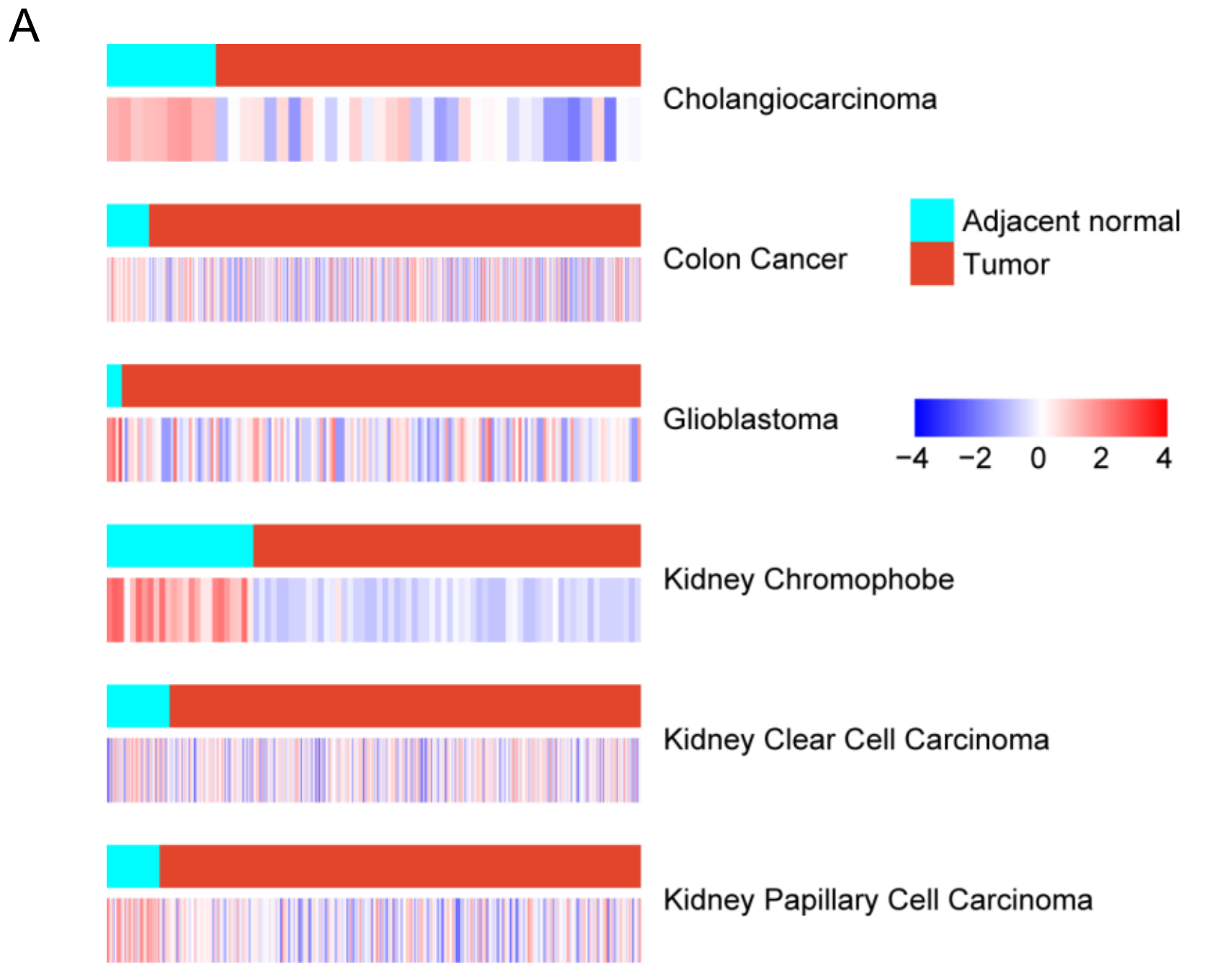
A
Cholangiocarcinoma
Colon Cancer
Glioblastoma
Kidney Chromophobe
Kidney Clear Cell Carcinoma
Kidney Papillary Cell Carcinoma
Adjacent normal
Tumor
−4 −2 0 2 4

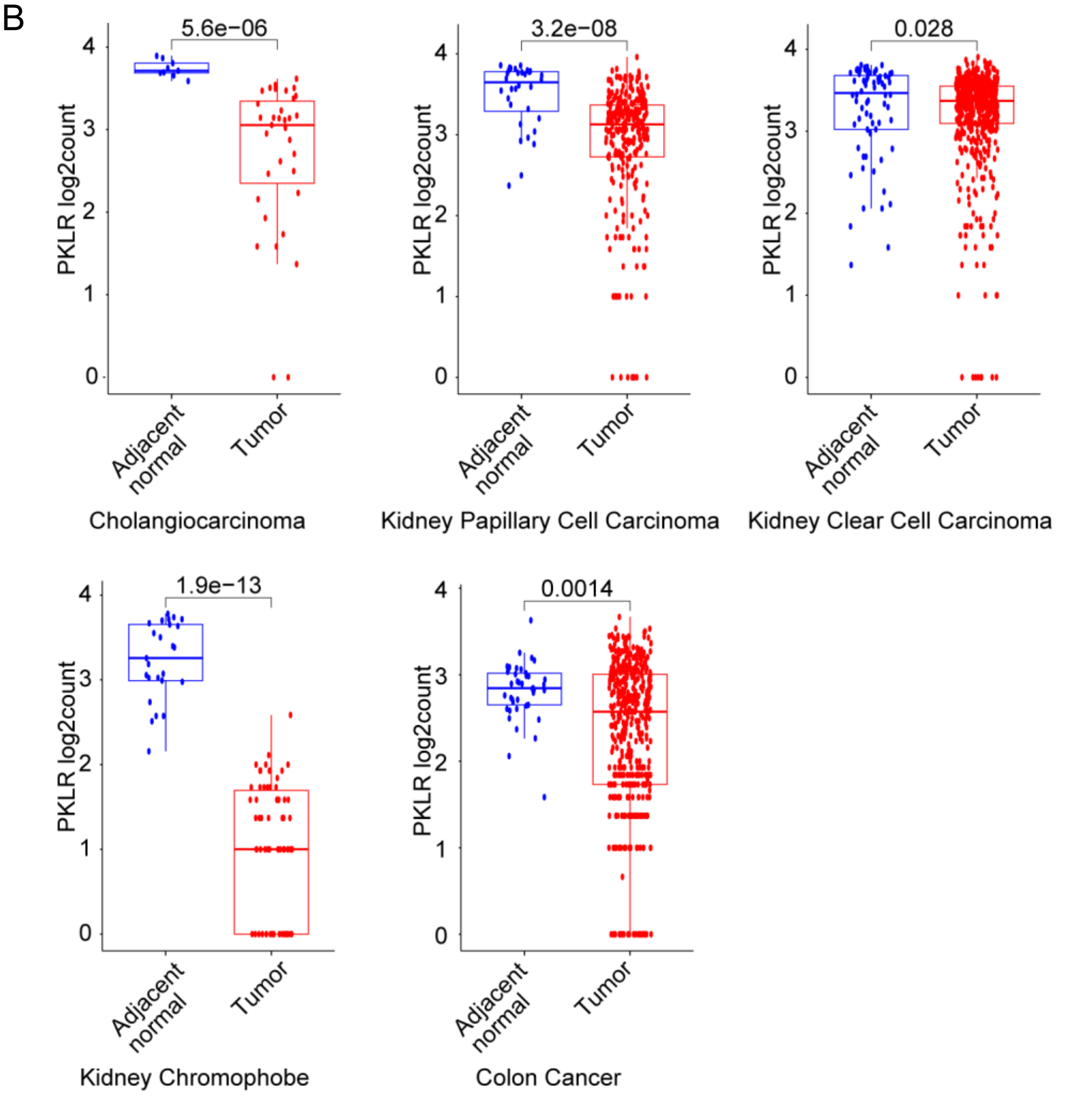
B
PKLR log2count
5.6e−06
3.2e−08
0.028
1.9e−13
0.0014
Adjacent normal
Tumor
Cholangiocarcinoma
Kidney Papillary Cell Carcinoma
Kidney Clear Cell Carcinoma
Kidney Chromophobe
Colon Cancer

Figure.S5

A

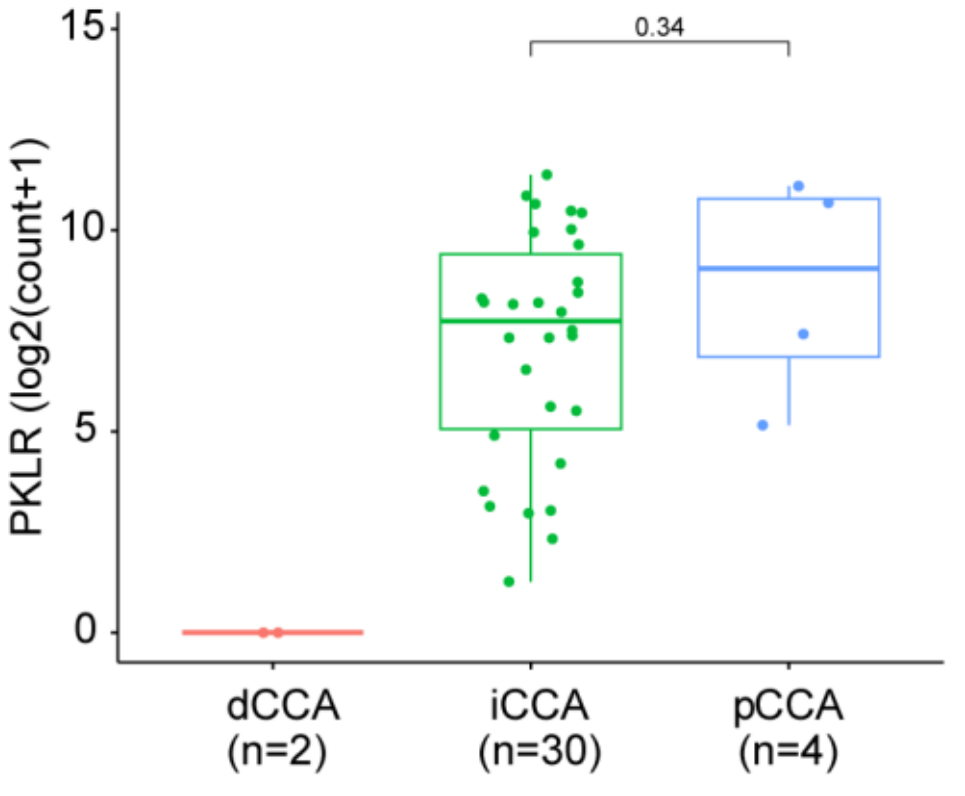

0.34
PKLR (log2(count+1))
15
10
5
0
dCCA
(n=2)
iCCA
(n=30)
pCCA
(n=4)

B

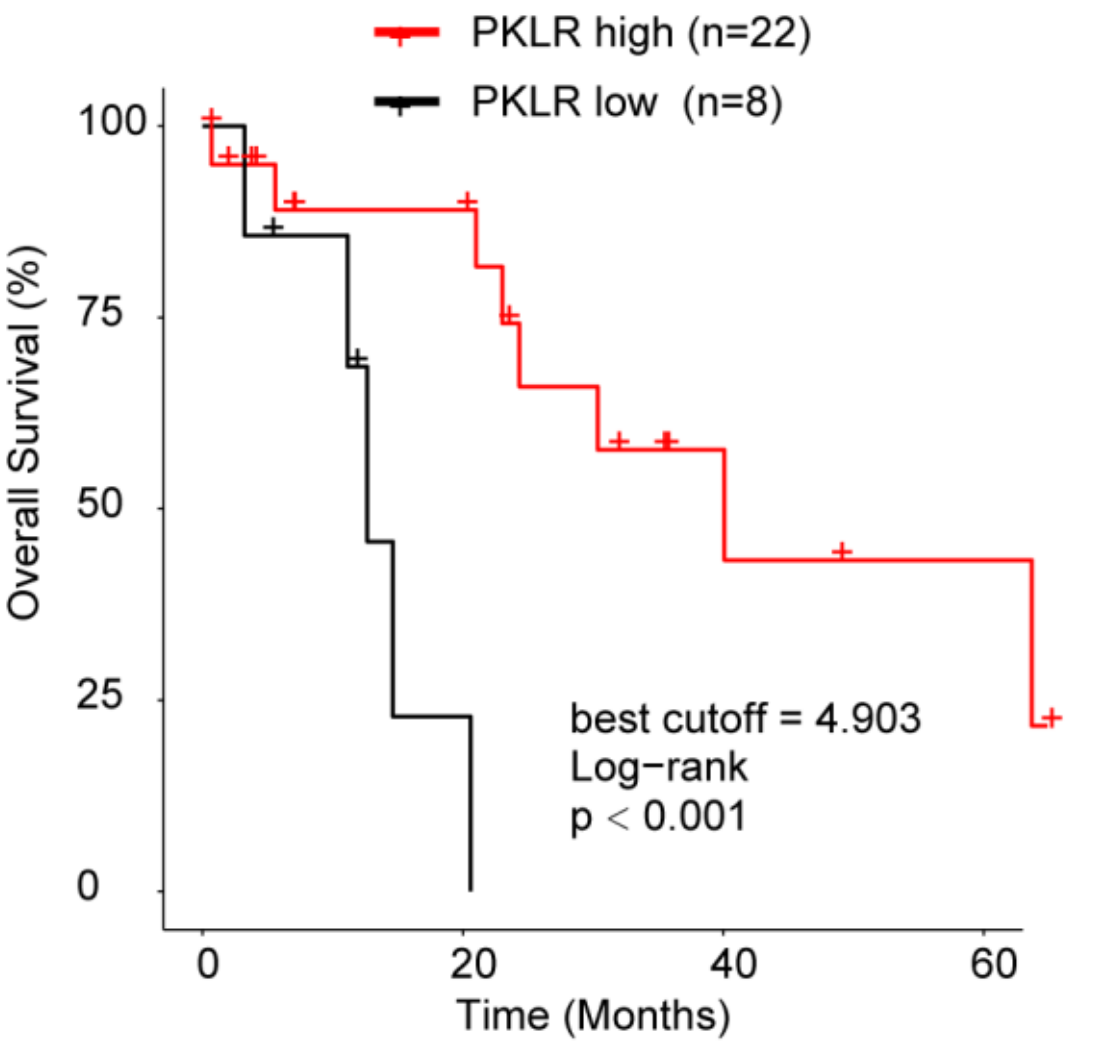

PKLR high (n=22)
PKLR low (n=8)
Overall Survival (%)
100
75
50
25
0
best cutoff = 4.903
Log−rank
p < 0.001
0
20
40
60
Time (Months)
