# Supplementary material for: Therapeutic and Predicted Significance of PKLR in Patients With Cholangiocarcinoma
Source: Cancer Rep (Hoboken). 2026 Aug 14;9(8):e70649. doi: 10.1002/cnr2.70649 (PMC13475273; doi:10.1002/cnr2.70649)
Supplement: Supplementary file 2 [file CNR2-9-e70649-s002.docx]

**Therapeutic and Predicted Significance of PKLR in patients with Cholangiocarcinoma**

**Supplementary methods**

**GEO (Gene Expression Omnibus) database**

RNA-seq data of GSE107943 (non-CCA: 27, CCA:30) and GSE26566 (adjacent normal:65, CCA:104) were downloaded from NCBI GEO database (https://www.ncbi.nlm.nih.gov/gds).

**Supplementary materials**

**Figure S1. The expression levels of** **the 10 potential prognostic biomarker and therapeutic targets between non-tumor tissues and CCA tumor tissues in GSE GSE26566 and** **GSE107943 cohorts.**

(A,) The expression levels of the 10 potential prognostic biomarker and therapeutic targets between non-tumor tissues and CCA tumor tissues in GSE107943 cohort (non-CCA: 27, CCA:30). (B, C) The expression levels of the 10 potential prognostic biomarker and therapeutic targets between non-tumor tissues and CCA tumor tissues in GSE26566 cohort (adjacent normal:65, CCA:104).

**Figure S2. The relationship of the 10 potential prognostic biomarker and therapeutic targets between mutated KRAS.**

(A) The relationship of the 10 potential prognostic biomarker and therapeutic targets between mutated KRAS in Fudan cohort. (B) The relationship of the 10 potential prognostic biomarker and therapeutic targets between mutated KRAS in TCGA cohort. Due to limited mutated KRAS patients’ number (n=2), statistics could not be performed on TCGA cohorts. * was <0.05, ** was <0.01, *** was <0.001. Error bar was calculated based on SEM.

**Figure S3.** **Differential expression of PKLR across 17 malignancies.**

(A) PKLR is upregulated in 6 tumor types relative to matched adjacent normal tissues. (B) PKLR expression shows no significant difference in 11 other tumor types.

**Figure S4. PKLR expression in malignancies originating from PKL-specific organs.**

(A, B) PKLR is downregulated in 6 tumor types relative to matched adjacent normal tissues, as shown by a heatmap (A) and a scatter plot (B).

**Figure S5. The expression level of PKLR among three subtypes of CCA in TCGA cohort.**

(A) Small size (n=2) of dCCA in TCGA cohort. And there was no significant expression level of PKLR between iCCA (n=30) and hCCA (n=4). (B) Kaplan-Meier curve shown OS of CCA patients between PKLR low and high groups based on TCGA iCCA patients. Best-cutoff value was re-calculated using R software.

| Table S1. Best cutoffs of 10 signatures using surv_cutpoint function in the R survminer package | | |
| --- | --- | --- |
| Symbols | Fudan | TCGA |
| FOLH1 | 3.274 | 245 |
| PKLR | 2.938 | 34.68 |
| GPR114 | 2.241 | 28 |
| KCNH2 | 0.163 | 121 |
| LCN2 | 5.825 | 8968 |
| MMP10 | 0.802 | 29 |
| PORCN | 4.8 | 284 |
| PTH1R | 2.028 | 97 |
| RIPK3 | 4.912 | 286 |
| SRPK3 | 1.346 | 57.14 |
| *All best cutoffs were shown as (log2(count+1) | | |

| **Table.S2** Univariate analyses of associations between overall survival and 10 potential signatures in the TCGA cohort (n=36) | | | | | |
| --- | --- | --- | --- | --- | --- |
| Univariable | patient’s numbers | log_2_HR | 95% C.I low | 95% C.I high | p value |
| FOLH1 | 8Versus.26 | -2.826 | -5.77 | 0.118 | **0.013** |
| PKLR | 24Versus.10 | -2.506 | -4.546 | -0.465 | **0.012** |
| GPR114 | 29Versus.5 | 2.712 | -0.24 | 5.664 | **0.02** |
| KCNH2 | 11Versus.23 | 1.606 | 0.065 | 3.147 | **0.046** |
| LCN2 | 6Versus.28 | 2.648 | 0.573 | 4.723 | **0.017** |
| MMP10 | 23Versus.11 | 2.126 | -0.02 | 4.272 | **0.022** |
| PORCN | 20Versus.14 | 1.835 | 0.106 | 3.565 | **0.028** |
| PTH1R | 20Versus.14 | -1.629 | -3.181 | -0.076 | **0.04** |
| RIPK3 | 21Versus.13 | 1.954 | 0.041 | 3.866 | **0.029** |
| SRPK3 | 10Versus.24 | 1.896 | 0.361 | 3.43 | **0.016** |

| **Table.S3** Univariate analyses of associations between overall survival and clinical features in thethe TCGA cohort (n=36) | | | | | |
| --- | --- | --- | --- | --- | --- |
| Univariable |  | log_2_HR | 95% C.I low | 95% C.I high | p value |
| Age (years old) | 63.028(29-82) | -0.627 | -2.183 | 0.929 | 0.442 |
| Gender(Male Versus. Female) | 16 Versus. 20 | -0.722 | -2.157 | 0.714 | 0.327 |
| AFP（ng/mL） | 3.753(1.38-14) | 0.052 | -0.188 | 0.291 | 0.682 |
| TB (μmol/L) | 0.679(0.2-2) | 0.121 | -2.632 | 2.875 | 0.932 |
| creatinine (μmol/L) | 0.859(0.5-1.4) | -0.162 | -3.294 | 2.97 | 0.919 |
| UICC T stage(T1 Versus.T2 Versus.T3) | 19 Versus. 12 Versus. 5 | 1.89 | -1.89 | -0.184 | 0.071 |
| N (N0 Versus.N1) | 26 Versus. 5 | 1.59 | -0.502 | 3.682 | 0.161 |
| M (M0 Versus. M1) | 28 Versus. 5 | 0.737 | -1.14 | 2.614 | 0.462 |
| Stage (I Versus. II Versus.III Versus.IV) | 19 Versus. 9 Versus. 1 Versus. 7 | 1.606 | 1.224 | -1.606 | 0.234 |
| Histological type (iCCA Versus. hCCA Versus. dCCA) | 30 Versus. 4 Versus. 2 | -26.068 | 26.068 | -0.947 | 0.337 |
| Race(Black/African American Versus. White Versus. Asian) | 2 Versus. 31 Versus. 3 | -0.981 | 0.981 | 1.728 | 0.586 |
| Pathological grading (G1 Versus. G2 Versus. G3 Versus. G4) | 1 Versus. 15 Versus. 18 Versus. 2 | 24.842 | 27.398 | -24.842 | **0.029** |
| Tumor status (with tumor Versus. tumor free) | 19 Versus. 15 | 3.396 | 0.463 | 6.329 | **0.002** |
| Resection Status (R0 Versus. R1) | 28 Versus. 5 | 1.948 | -0.079 | 3.975 | 0.086 |
| *For continuous variables, mean, minimum, maximum values were calculated. For categorical data, patient number were calculated. | | | | | |

| **Table.S4** Multivariate analyses of associations between overall survival and various risk factors in the TCGA cohort(n=36) | | | | | |
| --- | --- | --- | --- | --- | --- |
| Univariable |  | log_2_HR | 95% C.I low | 95% C.I high | p value |
| PKLR (High Versus. Low) | 24Versus.10 | -2.311 | -4.429 | -0.193 | **0.032** |
| (with tumor Versus. tumor free) | 19Versus.15 | 3.29 | 0.352 | 6.229 | **0.028** |
